# Supplementary material for: Observation of hyperbolic intersubband polaritons in native-dielectric-doped van der Waals semiconductor quantum wells
Source: Nat Commun. 2025 Nov 19;16:10158. doi: 10.1038/s41467-025-65196-y (PMC12630684; doi:10.1038/s41467-025-65196-y)
Supplement: Supplementary file 1 — Supplementary Information [file 41467_2025_65196_MOESM1_ESM.pdf]

Supplementary Information

Observation of hyperbolic intersubband polaritons in native-dielectric-doped van der Waals semiconductor quantum wells

Yue Luo[1,2,3*], Dapeng Ding[3,4], Andres M. Mier Valdivia[3], Daniel T. Larson[3], Song Liu[5], Hong Kuan Ng[3], Jing Wu[1], Kenji Watanabe[6], Takashi Taniguchi[7], Efthimios Kaxiras[3], Hongkun, Park[4], Philip Kim[3] and William L. Wilson[2*]

[1]School of Electronic Science and Engineering, Southeast University, Nanjing, Jiangsu, 210096, China

[2]Center for Nanoscale Systems, Harvard University, Cambridge, MA, 02138, USA

[3]Department of Physics, Harvard University, Cambridge, MA, 02138, USA

[4]Department of Chemistry and Chemical Biology, Harvard University, Cambridge, MA, USA

[5]Institute of Microelectronics, Chinese Academy of Sciences, Beijing, 100029, China

[6] International Center for Materials Nanoarchitectonics, National Institute for Materials Science, Namiki 1-1, Ibaraki 305-0044, Japan

[7] Research Center for Functional Materials Science, National Institute for Materials Science, Namiki 1-1, Ibaraki 305-0044, Japan

Correspondence to yueluo@seu.edu.cn and wwilson@cns.fas.harvard.edu

Supplementary Note 1: Self-limited oxidation of WSe_2.

We use UV-Ozone treatment to transfer the 5L- WSe_2 into WO_x/4L-WSe_2. This process is a self-limited oxidation process, where only the top most layer will be oxidized. As shown in **Supplementary Fig. 1a, b**, after 1 hour treatment, the optical contrast indicates only one layer of WSe2 transferred into WO_x. We further confirmed the result with atomic force microscope by taking line profile across the same flake before and after the oxidation process (**Supplementary Fig. 1c**). The layer thickness increased by about 0.8 nm. For the focused ion beam (FIB) fabrication, we perform UV-Ozone treatment after the FIB.

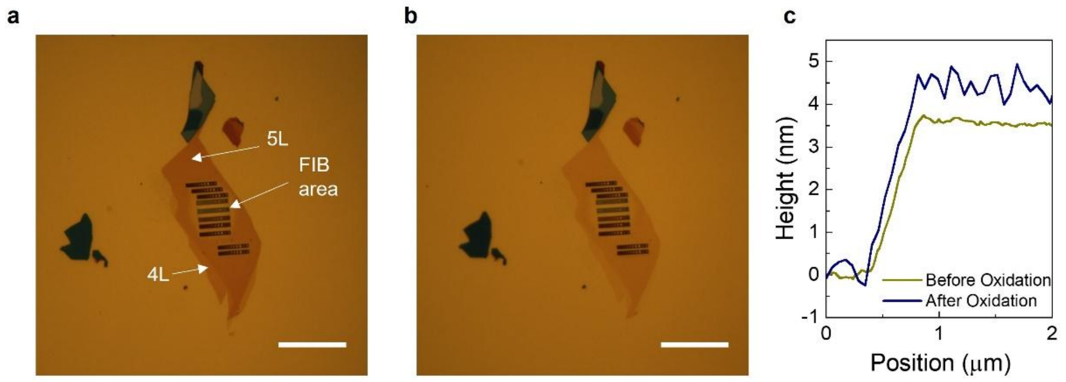

Supplementary Figure 1. Oxidation of WSe_2 with UV-Ozone treatment. a and **b**, Optical images of the same flake before (a) and after (b) the UV-Ozone treatment. Scale bar: 10 μm. **c**, Atomic force microscope height profile of the same 5L-WSe_2 flake before (blue solid lines) and after (green solid lines) the UV-Ozone treatment taken at the edge of the flake.

Supplementary Note 2: Field effect density

To determine the carrier density in the heterostructure, hole density in the *p*-doped WSe_2 multilayers is measured under ambient conditions using standard four-probe measurements. **Supplementary Figure 2** displays the optical microscope image of the device and the calculated hole density as a function of the contact bias.

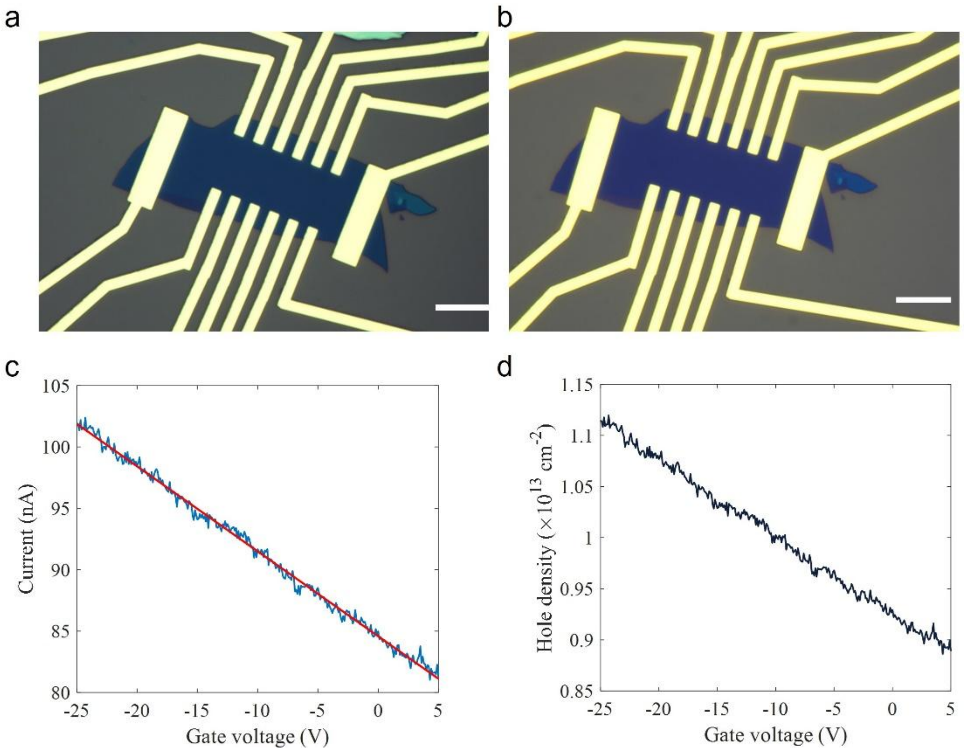

Supplementary Figure 2. **a** and **b**, Transport device optical images (a) before oxidation (b) after oxidation. The metal electrodes consist of 20 nm Pd / 70 nm Au. White scale bar: 10 μm. **c**, Current versus gate voltage at fixed voltage bias of 0.25 V. Blue curve: raw data. Red line: linear fit. The extracted slope is -0.69 nA/V. **d**, Hole density versus gate, calculated using the relation $\frac{IC}{e}\left(\left|\frac{dI}{dV_{Si}}\right|\right)^{-1}$.

Supplementary Note 3: Density function theory calculation of the electronic band structure and dielectric functions.

Density function theory (DFT) calculations including spin-orbit coupling were performed with the VASP package[1,2] using the Perdew-Burke-Ernzerhof exchange-correlation functional[3], the DFT-D3 method (zero damping) to account for van der Waals forces between layers[4], projector augmented wave (PAW) pseudopotentials with 14, 6, and 6 valences electrons for W, Se, and O, respectively[5,6], and a plane-wave energy cutoff of 500 eV. Momentum space was sampled with a 12×12×1 Γ-centered grid.

The crystal surface was modeled using a slab geometry with a single WSe_2 unit cell in the horizontal plane, 5 layers following 2H stacking in the z-direction, and at least 26 Å of vacuum space. The horizontal lattice constants were optimized for a bulk cell and then kept constant for the slab structures, where the ionic positions were relaxed to a tolerance of 1 meV/Å. To study the effect of oxidation, the Se atoms in the topmost layer were replaced with oxygen atoms and the ionic positions were re-relaxed to the same tolerance. Note that the WSe_2 lattice constant of ~3.3 Å is significantly larger than the optimized lattice constant of ~2.8 Å for monolayer WO_x. Thus, the WO_x layer in the slab model is significantly expanded from its nominal horizontal lattice constant. By fitting to the dispersion near the valence band edge with $E = -\hbar^2 k^2/2m^*$, we found that the in-plane effective mass $m^* = 0.91\ m_0$, where m_0 is the free electron mass, which is close to the literature value[7].

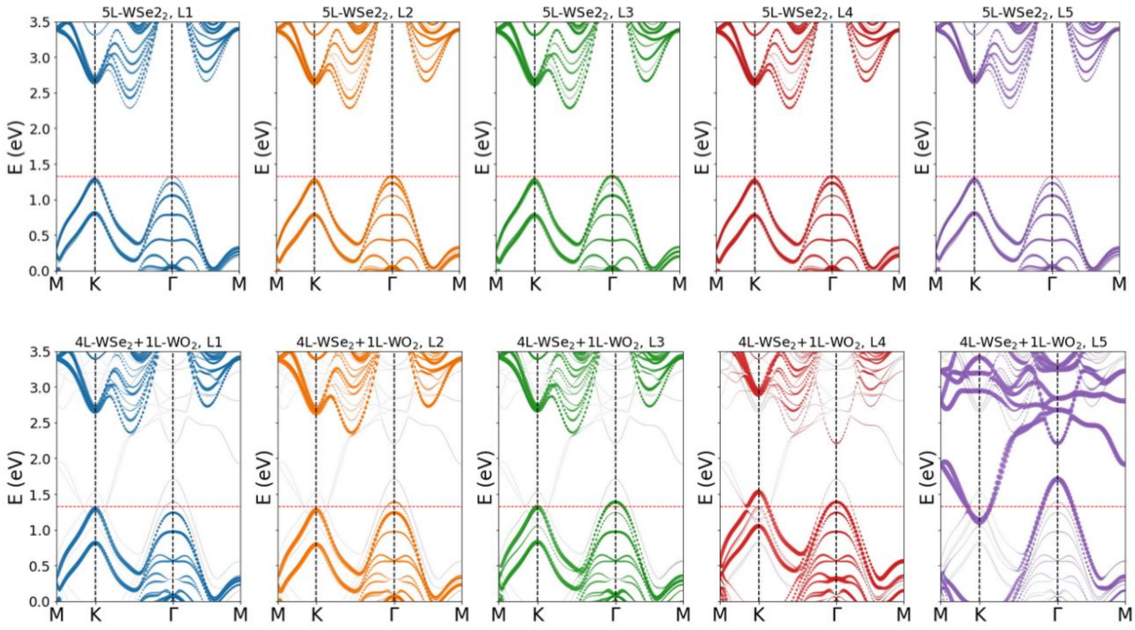

Supplementary Figure 3. DFT calculated band structure of layer projection for the 5L-WSe_2 (top) and WO_x/4L-WSe_2 (bottom) showing contribution from each physical layers.

We also studied the relation between the physical layer and the subband. If the Fermi level crosses several parabolic bands, then the band with highest energy will have the highest fraction unoccupied, and thus the highest carrier concentration. Note that the highest band does not

necessarily originate from the top physical layer. **Supplementary Figure 3** presents the layer-projected densities of states for the 5-layer (5L) WSe_2 slab. In the pristine 5L-WSe_2 structure, which retains its symmetry, the top valence subband primarily originates from the middle layer (L3), with some contribution from the adjacent layers (L2 and L4). The second valence subband is predominantly derived from layers L2 and L4, while the third valence subband arises mainly from layers L1, L3, and L5. However, upon oxidation of the top layer (L5), the symmetry is broken. In this case, the top valence subband shifts to predominantly arise from the oxidized top layer (L5). Subsequently, the second valence subband becomes primarily associated with layers L3 and L4, and the third valence subband is largely attributed to layer L2.

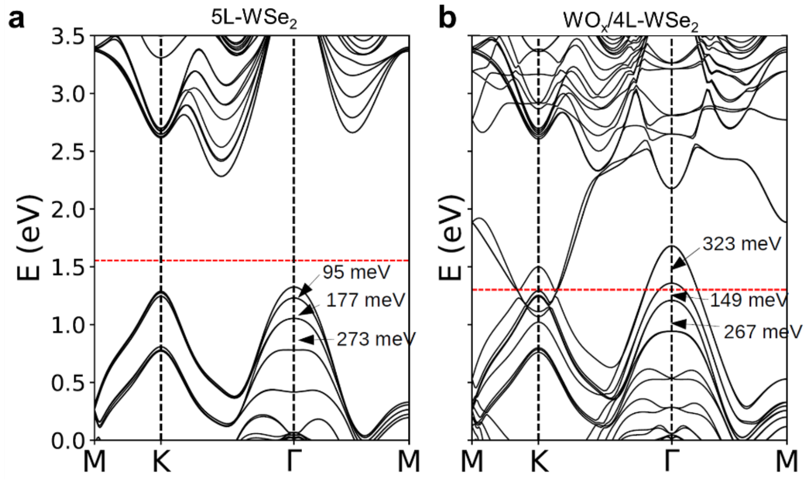

Supplementary Figure 4. **a** and **b**, Band structure comparison before (a) and after oxidation process.

Supplementary Note 4: Model of the dielectric tensor for WO_x/4L-WSe_2 heterostructure.

The effective dielectric response tensor of WO_x/4L-WSe_2 heterostructure can be written as:

$\varepsilon_{\text{eff}} = \begin{bmatrix} \varepsilon^{xx} & 0 & 0 \\ 0 & \varepsilon^{yy} & 0 \\ 0 & 0 & \varepsilon^{zz} \end{bmatrix}$, where $\varepsilon^{xx} = \varepsilon^{yy}$ are the in-plane components of the dielectric tensor and ε^{zz} is the out-of-plane component of the dielectric tensor. The real part of the in-plane components ε^{xx} and ε^{yy} are feature less in this mid-infrared range with no absorption. The static permittivity for intrinsic WSe_2 is take from literature as $\varepsilon^{\infty,xx} = \varepsilon^{\infty,yy} = 13.7$, and $\varepsilon^{\infty,zz} = 4.0$ We model the out-of-plane component ε^{zz} with Drude-Lorentz model: $\varepsilon(\omega) = \varepsilon_\infty - \frac{\omega_p^2}{\omega^2 + i\omega\gamma_D} - \frac{Ne^2}{m^*\varepsilon_0}\frac{f}{\omega_0^2 - \omega^2 - i\gamma_L\omega}$, where ε_∞ is the high-frequency dielectric permittivity, ω_0 is the oscillator frequency, N is the hole density, ε_0 and e are the fundamental constants of the permittivity of free space and electron charge. f is the Lorentz oscillator strength and γ_L is the linewidth of the Lorentz oscillator.

We then fit the nano-FTIR spectra using the finite dipole model (FDM), where the tip is represented with an ellipsoid to fit the nano-FTIR data[8,9]. By demodulating the signal at higher harmonics of the probe vibration frequency Ω, the pure near-field signal can be extracted from the total scattered field. The nth harmonic σ_n of the scattering coefficient σ can be calculated as the nth Fourier series coefficient of σ with respect to the probe vibration frequency Ω over the fundamental period $T = 2\pi/\Omega$: $\sigma_n = \frac{1}{T}\int_{-T/2}^{T/2} \sigma(t) e^{-in\Omega t} dt$. The effective polarizability of the tip-sample system under the excitation field is given by $\alpha_{\text{eff}} \propto \alpha_{\text{tip}}(1+\beta)(1 - \frac{\alpha_{\text{tip}}\beta}{16\pi h^2})$, where h is the tip-sample distance, $\alpha_{\text{tip}}$is the polarizability of the tip and $\beta = \frac{\varepsilon - 1}{\varepsilon + 1}$ is the optical response from the sample[8,10]. The tip polarizability $\alpha_{\text{tip}} = 4\pi r^3(\varepsilon_{\text{tip}} - 1)/(\varepsilon_{\text{tip}} + 2)$. Here the tip radius is r = 20 nm, tip sample distance h = 60 nm and ε_{tip} is a large negative number at IR frequency. We fitted the nano-FTIR spectra of WO_x/4L-WSe_2 heterostructure and shown in **Supplementary Fig. 5**. For the phase spectrum, the peak position and FWHM are the key properties that indicate the absorption of the ISB. The anisotropic parameter can be expressed as $\kappa = \frac{\varepsilon^{\infty,zz} m^{zz}}{\varepsilon^{\infty,xy} m^{xy}} \cong 0.5$, suggesting strong anisotropy. We find the center energy of 129 meV and full-with half-maximum of the absorption peak Γ = 5.27 meV at room-temperature. The Lorentz oscillator strength of f = 1.2 is used as fitting parameter. For the amplitude spectrum, the falling edge is the key feature.

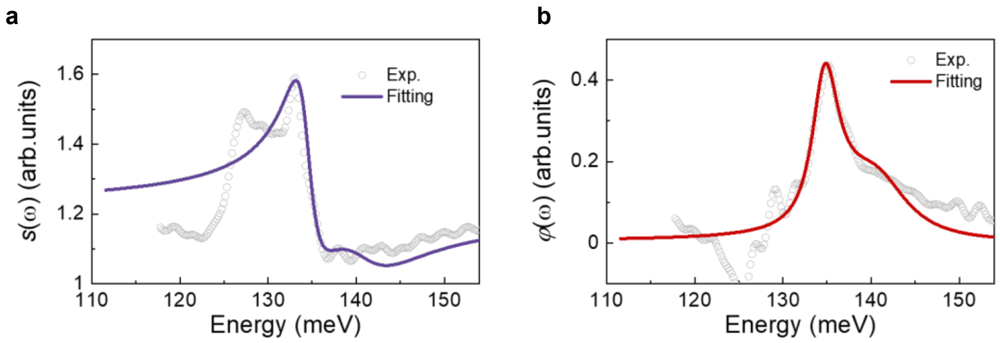

Supplementary Figure 5. Fitting of the nano-FTIR spectrum with finite dipole model. a, Experimental (gray circles) and dipole model fitting (purple solid lines) of the near-field amplitude $s(\omega)$. **b**, Experimental (gray circles) and dipole model fitting (red solid lines) of the near-field amplitude $\varphi(\omega)$.

Supplementary Note 5: Propagation length and quality factor of ISP.

In order to calculate the quality factor of ISP, we first extract the propagation length from the near-field amplitude image. We quantitatively analyze the ISP propagation by fitting the s-SNOM amplitude line profile with an exponentially decaying sinusoidal function: $y = Ae^{-x/L_x}\sin(2\pi x/\rho - B) + C$, where L_x is the decay length, and A, B, and C are fitting parameters. The experimental data is corrected by a factor of $\sqrt{x}$ to compensate the geometrical decay. During the fitting process, we achieved convergence criteria using the Levenberg-Marquardt algorithm. A representative fitting to the line profile taken at E_{ph} = 144 meV is shown in **Supplementary Fig. 6**. Propagation length L_x is fitted to be 0.8 µm.

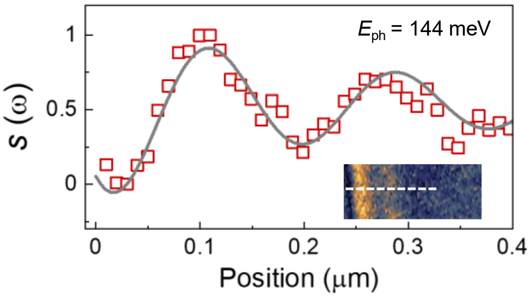

Supplementary Figure 6. Fitting of line profile with exponential decay function.

For comparison, we have also checked our fitting with complex-valued analysis[11]. The complex-valued line profile can be expressed as $\sigma(x) = s(x)e^{i\varphi x}$ and plotted as trajectories in the complex plane (**Supplementary Figure 7a**). We then fit the experimental data points with a damped spiral function $E_p = Ae^{i2q_{ISP}x}/\sqrt{2x}$+C, where A and C are fitting parameters. We then fit the amplitude of the line profile which is the real-part of $\sigma(x)$ using the exponentially decaying sinusoidal function with the extracted q_{ISP} from E_p (**Supplementary Figure 7b**). We find agreement between the two fitting processes. The difference between the complex-valued analysis and exponentially decaying sinusoidal function is relatively small when the number of fringes is more than one.

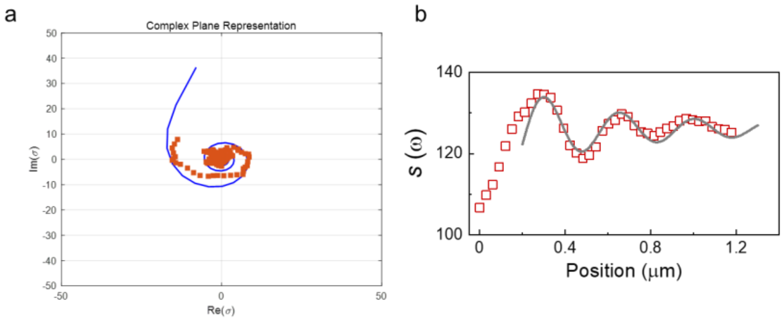

Supplementary Figure 7. Fitted line-profile with complex- valued analysis (a) and exponentially decaying sinusoidal function (b).

Supplementary Note 6: Intersubband polariton dispersion calculation.

We calculate the ISP dispersion using the dielectric function of WO_x/4L-WSe_2 heterostructure. We can solve the eigenequation of the out-of-plane ISP $\sqrt{\varepsilon_z k_0^2 - \beta_z^2}d = tan^{-1}\left(\frac{\sqrt{\beta_z^2 - k_0^2\varepsilon_1}}{\sqrt{\varepsilon_z k_0^2 - \beta_z^2}}\right) + tan^{-1}(\frac{\sqrt{\beta_z^2 - k_0^2\varepsilon_2}}{\sqrt{\varepsilon_z k_0^2 - \beta_z^2}}) + m\pi$. The effective dielectric permittivity of the heterostructure is adopt using a Lorentz model mentioned in **Supplementary Note 4**. The s-SNOM measured scattered field can be approximated as the complex reflectivity $r_{\mathrm{ISP}}(k,\omega)$, which is extended studied in the polariton systems such as surface plasmon in graphene and phonon polariton in h-BN[12]. In our system, for simplicity, we only consider two interfaces: Air/ WO_x-WSe_2 and WO_x-WSe_2/SiO_2. Using Fresnel reflection coefficients for the i-j interface: $r_{ij} = \frac{Q_j - Q_i}{Q_j + Q_i}, Q_j = \frac{\varepsilon_j}{k_j}$. So the $r_{\mathrm{ISP}} = \frac{r_{01} + r_{12}e^{2i\beta d}}{1 + r_{01}r_{12}e^{2i\beta d}}$. The layer thickness of WO_x/4L-WSe_2 heterostructure is d = 4.5 nm. We plot the dispersion relation, $|Im(r_{\mathrm{ISP}})|$ and experimental data points. The calculated dispersion relation matches the experimental data well as shown in **Fig 2f** of the main text.

Supplementary Note 7: Layer number dependence of ISP.

The transition energy is determined by the crystal layer structure. We have studied the ISP with different layer thickness. **Supplementary Figure 8a** shows DFT calculation of the band structure for 3 to 6 layers WSe_2. The intersubband energy decreases as the layer number increasing. Considering the limited accessible laser energy range, we fabricated WO_x/6L-WSe_2 heterostructure which should have a ISB energy of 113meV according to our calculation. **Supplementary Fig.8b** shows near-field nano-image of the heterostructure with excitation energy E_{ph}=117meV. We observed interference fringe at the flake boundary and analyzed the fringe periodicity by assuming the phase change at the flake edge (**Supplementary Fig.8c**). By changing the excitation energy, we find a negative dispersion with a lower transition energy, which matches the calculated value of 113 meV. As the layer number increasing, our DFT calculation indicates that the subband transition energy decreases. Our data suggested a clear layer number dependence agree with the nature of the ISB transition.

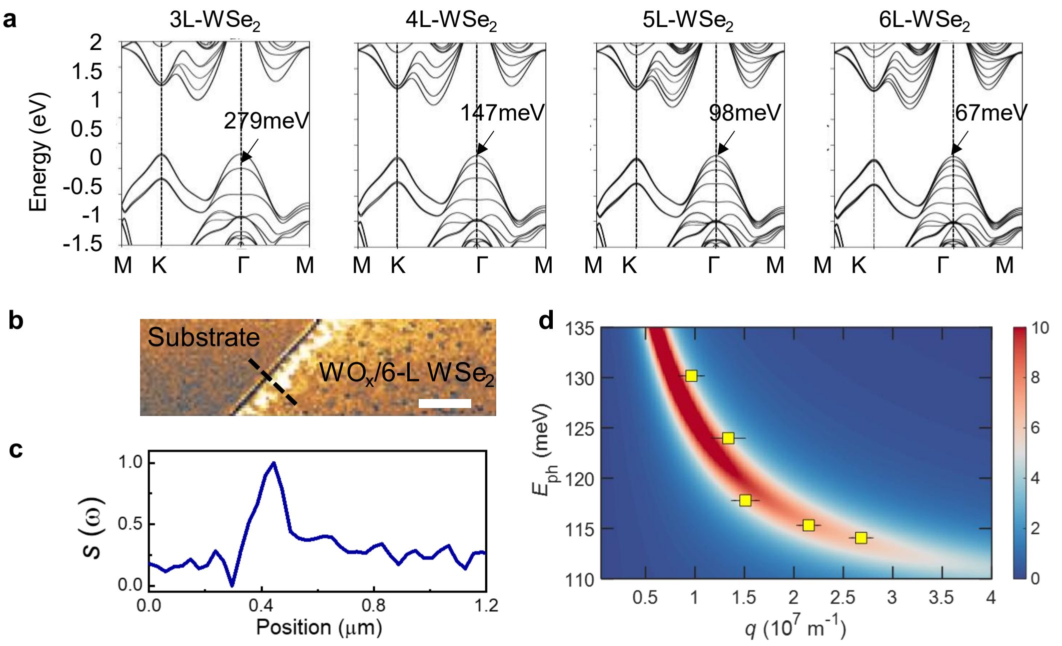

Supplementary Figure 8. **a,** DFT calculated band structure of 3 to 6 layers of WSe_2. **b,** Near-field amplitude image of the ISP in WO_x/6L WSe_2. **c**, Line profile taken at the sample edge. **d**, Dispersion of the ISP in WO_x/6L-WSe_2. Color plot is calculated using the Fresnel reflection coefficients Im(r_p). Yellow squares are experimental data.

Supplementary Note 8: Gate tunable ISPs

The data points in Fig.2f are experimental results extracted from the line profiles taken at different gate voltages (**Supplementary Figure 9**).

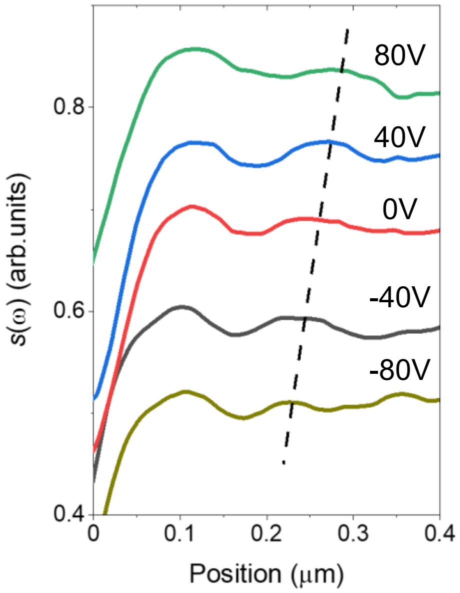

Supplementary Figure 9. Extracted line profiles at different gate voltages. Black dashed line help to visualize the polariton wavelength shift.

Supplementary Note 9: Prolonged propagation length using Au mirror

Radiative decay into the silicon substrate constitutes one of the main loss channels for the ISP. To suppress this loss and enhance the visibility of interference fringes, we transferred the WO_x/5L-WSe_2 heterostructure onto a 100-nm-thick Au mirror. As shown in **Supplementary Fig. 10**, this configuration yields a larger number of fringes with improved contrast. The real-part of the complex momentum is $q'_{\mathrm{ISP}} = 2\pi/\lambda_{\mathrm{ISP}}$ and the imaginary-part of the momentum is related to the propagation length as $q''_{\mathrm{ISP}} = 1/(2L)$. We find $Q \sim 16.9$.

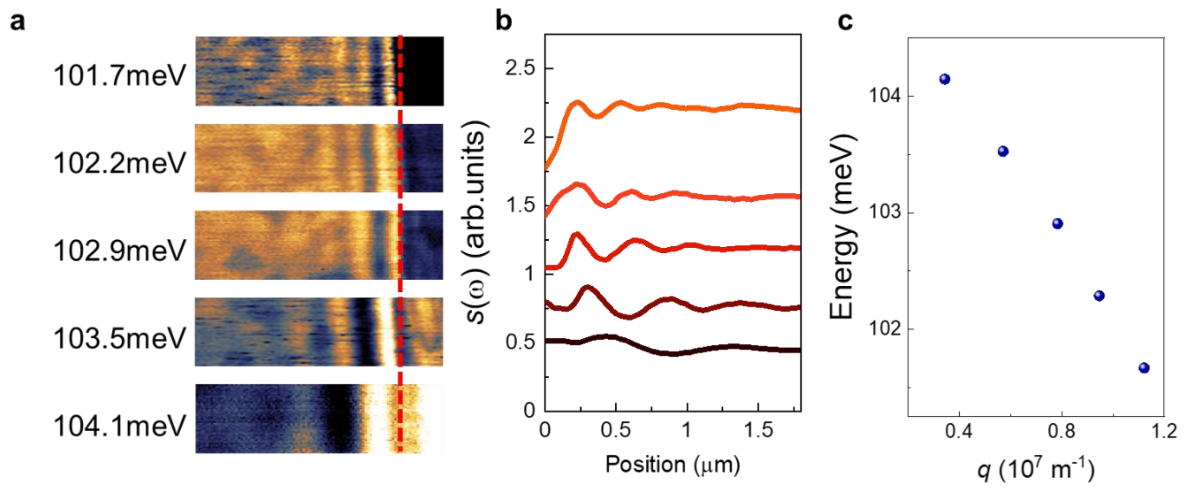

Supplementary Figure 10. a, Nano-imaging of the WO_x/5L-WSe_2 heterostructure on the Au mirror at various excitation energies. Red dashed line indicates the flake boundary. **b**, Line-profiles extracted at each excitation energies. **c**, Experimental dispersion relation calculated from the fringe periodicity.

Supplementary Note 10: Lifetime for ISP.

The lifetime of the ISP polariton can be calculated using $\tau_{\mathrm{ISP}} = L/v_g$, where L is the propagation length and v_g is the group velocity. The propagation length is extracted from the fitting parameter of the line profile while v_g is calculated using the dispersion relation $v_g = \frac{1}{\hbar}\frac{dE}{dk}$. Supplementary Figure 10 shows v_g as a function of E, where we found v_g=1.71×10^6 m/s or 0.0057c at E=0.144eV. Therefore, the lifetime is $\tau_{\mathrm{ISP}} = \frac{L}{v_g} = \frac{0.8\times10^{-6}}{1.71\times10^{6}} = 0.468\,ps$ for WO_x/4L-WSe_2 on the SiO_2/Si substrate. When using the Au substrate, $\tau_{\mathrm{ISP}} = \frac{1.3\times10^{-6}}{5\times10^{5}} = 2.6ps$ for WO_x/5L-WSe_2.

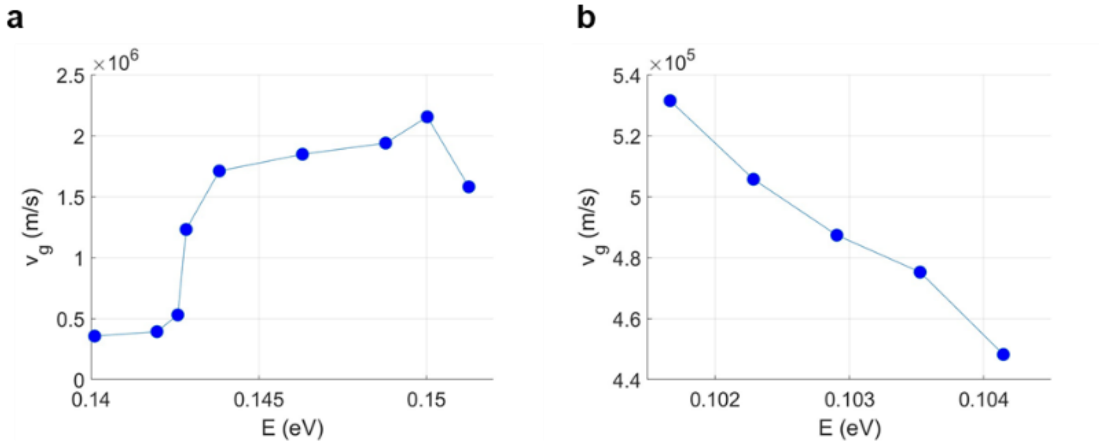

Supplementary Figure 11. Group velocities for WO_x/4L-WSe_2 (a) and WO_x/5L-WSe_2 (b).

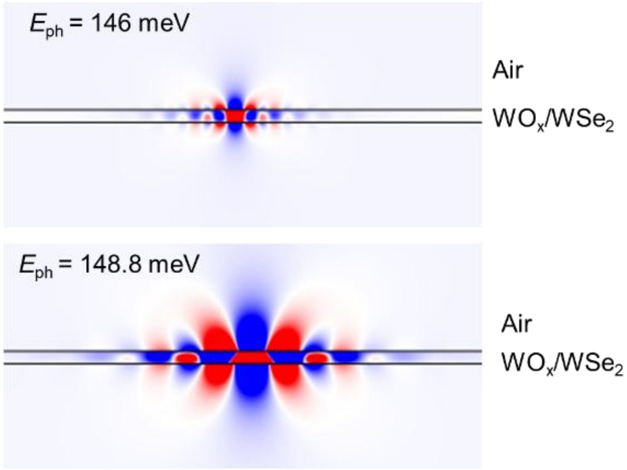

Supplementary Figure 12 Simulated field profiles for the WO_x/WSe_2 heterostructure at different excitation energies that show the fundamental mode.

Supplementary References

1. Kresse, G. & Furthmüller, J. Efficient iterative schemes for ab initio total-energy calculations using a plane-wave basis set. *Phys. Rev. B* **54**, 11169 (1996).
2. Kresse, G. & Furthmüller, J. Efficiency of ab-initio total energy calculations for metals and semiconductors using a plane-wave basis set. *Comput. Mater. Sci.* **6**, 15–50 (1996).
3. Perdew, J. P., Burke, K. & Ernzerhof, M. Generalized gradient approximation made simple. *Phys. Rev. Lett.* **77**, 3865 (1996).
4. Grimme, S., Antony, J., Ehrlich, S. & Krieg, H. A consistent and accurate ab initio parametrization of density functional dispersion correction (DFT-D) for the 94 elements H-Pu. *J. Chem. Phys.* **132**, 154104 (2010).
5. Blöchl, P. E. Projector augmented-wave method. *Phys. Rev. B* **50**, 17953 (1994).
6. Kresse, G. & Joubert, D. From ultrasoft pseudopotentials to the projector augmented-wave method. *Phys. Rev. B* **59**, 1758 (1999).
7. Schmidt, P. *et al.* Nano-imaging of intersubband transitions in van der Waals quantum wells. *Nat. Nanotechnol.* **13**, 1035–1041 (2018).
8. Chui, S. T. *et al.* Effect of sample anisotropy on scanning near-field optical microscope images. *J. Appl. Phys.* **129**, 083105 (2021).
9. Cvitkovic, A., Ocelic, N. & Hillenbrand, R. Analytical model for quantitative prediction of material contrasts in scattering-type near-field optical microscopy. *Opt. Express* **15**, 8550 (2007).
10. Kehr, S. C. *et al.* Anisotropy Contrast in Phonon-Enhanced Apertureless Near-Field Microscopy Using a Free-Electron Laser. *Phys. Rev. Lett.* **100**, 256403 (2008).
11. Chen, S. *et al.* Real-space nanoimaging of THz polaritons in the topological insulator Bi2Se3. *Nat. Commun.* **13**, (2022).
12. Dai, S. *et al.* Tunable Phonon Polaritons in Atomically Thin van der Waals Crystals of Boron Nitride. *Science* **343**, 1125–1129 (2014).
